# Glycosylation Inhibitors Efficiently Inhibit P-Selectin-Mediated Cell Adhesion to Endothelial Cells

**DOI:** 10.1371/journal.pone.0099363

**Published:** 2014-06-19

**Authors:** Pushpankur Ghoshal, Mythilypriya Rajendran, Nadine Odo, Tohru Ikuta

**Affiliations:** Department of Anesthesiology and Perioperative Medicine, Medical College of Georgia, Georgia Regents University, Augusta, Georgia, United States of America; University of Kentucky, United States of America

## Abstract

Adhesion molecules play a critical role in the adhesive interactions of multiple cell types in sickle cell disease (SCD). We previously showed that anti-P-selectin aptamer efficiently inhibits cell adhesion to endothelial cells (ECs) and permits SCD mice to survive hypoxic stress. In an effort to discover new mechanisms with which to inhibit P-selectin, we examined the role of glycosylation. P-selectin is a 90 kDa protein but was found to migrate as 90 and 140 kDa bands on gel electrophoresis. When P-selectin isolated from ECs was digested with peptide *N*-glycosidase F, but not *O*-glycosidase, the 140 kDa band was lost and the 90 kDa band was enhanced. Treatment of ECs with tunicamycin, an *N*-glycosylation inhibitor, suppressed CD62P (P-selectin) expression on the cell surface as well as the 140 kDa form in the cytoplasm. These results indicate that the 140 kDa band is *N*-glycosylated and glycosylation is critical for cell surface expression of P-selectin in ECs. Thrombin, which stimulates P-selectin expression on ECs, induced AKT phosphorylation, whereas tunicamycin inhibited AKT phosphorylation, suggesting that AKT signaling is involved in the tunicamycin-mediated inhibition of P-selectin expression. Importantly, the adhesion of sickle red blood cells (sRBCs) and leukocytes to ECs induced by thrombin or hypoxia was markedly inhibited by two structurally distinct glycosylation inhibitors; the levels of which were comparable to that of a P-selectin monoclonal antibody which most strongly inhibited cell adhesion in vivo. Knockdown studies of P-selectin using short-hairpin RNAs in ECs suppressed sRBC adhesion, indicating a legitimate role for P-selectin in sRBC adhesion. Together, these results demonstrate that P-selectin expression on ECs is regulated in part by glycosylation mechanisms and that glycosylation inhibitors efficiently reduce the adhesion of sRBCs and leukocytes to ECs. Glycosylation inhibitors may lead to a novel therapy which inhibits cell adhesion in SCD.

## Introduction

Sickle cell disease (SCD) is caused by a mutation in the β-globin gene that replaces glutamic acid with valine. The resulting sickle hemoglobin polymerizes from a variety of physiologic insults such as infection and hypoxia [1]. Ever since the molecular basis of this disorder was clarified [2], considerable effort has been directed toward developing therapeutics to alleviate the clinical severity of SCD [3]. Fetal hemoglobin inhibits sickle hemoglobin polymerization in vitro [4] and is an important protein ameliorating disease severity [5], as evidenced by the fact that SCD patients who express high levels of fetal hemoglobin have a milder clinical course [6]. Multiple clinical studies have shown that hydroxyurea, an S stage-specific chemical that was approved to treat SCD [7], increases fetal hemoglobin levels in SCD patients and alleviates clinical severity [8], [9].

While fetal hemoglobin induction is a critical parameter in evaluating the clinical effectiveness of hydroxyurea, it could be argued that reducing the frequency of vaso-occlusive crises [10], the hallmark manifestation of SCD, may be more germane. Current clinical management of vaso-occlusive crises largely relies on palliative therapies including opioids and non-steroidal anti-inflammatory agents [11]. To gain insight into the molecular and physiological mechanisms underlying vaso-occlusive crisis, a number of adhesion molecules on multiple cell types have been identified by various in vitro experimental systems. Adhesion molecules identified thus far include vascular cell adhesion molecule-1 (VCAM-1) [12], selectins [13], [14], laminin [15], thrombospondin [16], fibronectin [17], and αvβ3 integrin [18]. Selectins in particular have been implicated in the adhesive interactions of sRBCs and leukocytes with ECs by intravital microscopy [19], [20]. Our intravital microscopic studies found that anti-P-selectin aptamer, with its high affinity to P-selectin and inhibition of P-selectin function, enables SCD model mice to survive hypoxic stress [21]. This is consistent with the work by Embury and colleagues which revealed an important role for P-selectin in sRBC adhesion to ECs [13], [22]. Like anti-P-selectin aptamer, low-molecular-weight heparin (LMWH) is a strong P-selectin inhibitor and another candidate for preventing vaso-occlusive crisis in SCD [14]. A recent phase II clinical trial of pentosan polysulfate sodium (PPS), an orally available heparin compound, improved microvascular flow and reduced serum VCAM-1 levels in SCD patients, but did not reduce daily pain scores [23], prompting us to search for novel P-selectin inhibitors.

To identify more potent and efficacious P-selectin inhibitors, in this study we focused on the molecular mechanisms by which P-selectin expression is regulated on the cell surface of ECs. Previous studies showed that P-selectin is a highly glycosylated protein with the molecular weight of 90 kDa [24]. We investigated the effect of glycosylation inhibitors on P-selectin expression on ECs as well as on sRBC and leukocyte adhesion to ECs. We found that glycosylation inhibitors efficiently inhibit P-selectin expression on ECs by interfering with glycosylation processes but without affecting mRNA expression and proteasomal degradation of the protein. In this way, glycosylation inhibitors reduce the adhesion of both sRBCs and leukocytes to ECs. Importantly, the degree to which glycosylation inhibitors limit sRBC adhesion to ECs was comparable to that of an anti-P-selectin monoclonal antibody which we had previously studied [21]. To our knowledge, this is the first study to demonstrate the ability of glycosylation inhibitors to limit sRBC and leukocyte adhesion to ECs. A number of orally available glycosylation inhibitors are being tested in multiple clinical studies for various disorders [25], and such inhibitors, alone or in combination with other P-selectin inhibitors, may offer a novel means of inhibiting vaso-occlusive crisis in SCD.

## Materials and Methods

### Peripheral Blood from SCD Patients and SCD Model Mice

Peripheral blood was obtained from SCD patients who were homozygous for the β^S^ mutation. The study was performed in accordance with the principles of the Declaration of Helsinki and approved by the Georgia Regents University institutional review board. Informed written consent form was obtained from all subjects. Peripheral blood was also collected from knockout-transgenic sickle cell mice [26]. The animal study was approved by the Institutional Animal Care and Use Committee of Georgia Regents University.

### In vitro Culture of HUVECs

Primary human umbilical vein endothelial cells (HUVECs) (CRL #1730, ATCC, Manassas, VA) were cultured according to the manufacturer’s instructions in a vascular basal cell medium supplemented with a bovine brain extract endothelial cell growth kit at 37°C and 5% CO_2_. Cells were maintained in a serum-free medium for 16 hours before experiments were performed. To expose cells to hypoxic stress, cells were maintained at a constant gas mixture of 1% O_2_, 94% N_2_, and 5% CO_2_ in a CO_2_ incubator equipped with an O_2_ sensor (Fisher Scientific, Waltham, MA).

### Western Blot Analysis

Western blotting was performed as previously described [27]. Briefly, protein lysates were prepared by incubating cells with a lysis buffer as described [27]. Cell debris was removed by centrifugation (14,000×g, 15 min at 4°C). To isolate membrane proteins, we used the Mem-PER Plus Membrane Protein Extraction Kit (Pierce, Rockford, IL). Cells were first permeabilized with a mild detergent to release soluble cytosolic proteins, centrifuged at 16,000×g, then treated with a second detergent to solubilize membrane proteins. Proteins (50 µg) were resolved in 12% Tris-acetate gel (BioRad, Hercules, CA) and transferred onto polyvinylidene difluoride membranes (GE Healthcare, Pittsburgh, PA) using a transfer system (Bio-Rad). The membranes were probed with anti-P-selectin (Santa Cruz Biotechnology, Dallas, TX), P-AKT antibodies (Cell Signaling, Danvers, MA) or β-actin (Santa Cruz), followed by corresponding secondary antibodies. Western blots were developed using the ECL Plus Western blot detection kit (BioRad).

### Digestion of P-selectin with Glycosidases

Cell lysates were prepared from HUVECs stimulated with thrombin (1 U/mL). One milligram of total cell lysates was immunoprecipitated using P-selectin antibody (H-150) and protein A agarose beads as described [27]. Proteins immobilized on agarose beads were eluted with 0.1 M glycine buffer (pH 2.8). Eluted proteins were then treated with 1000 units of PNGase F or 4×10^6^ units *O*-glycosidase (New England Biolabs, Ipswich, MA) at 37°C in the reaction buffer containing 50 mM sodium phosphate, pH 7.5, 0.5% SDS, 40 mM DTT, and 1% NP40 as described [28]. The resultant products were analyzed by Western blotting with anti-P-selectin antibody.

### Flow Cytometry

Cells were treated in PBS containing 2 mM EDTA and 1 U/mL thrombin for 10 minutes at room temperature. Paraformaldehyde was added in an equal volume to a final concentration of 1% to fix the cells for 30 minutes at room temperature. The cells were then washed twice in PBS containing 0.1% sodium azide and 1% fetal bovine serum (FBS) and stained with a phycoerythrin (PE)-labeled anti-human P-selectin monoclonal antibody (CD62P PE) (eBioscience, San Diego, CA) or a matching isotype control at 4°C for 30 minutes followed by a Perm/Wash step as described in the manufacturer’s protocol. CD 59 FITC (eBioscience) was used as a positive marker for EC. Data were represented as percent of positive cells.

### RNA Extraction and PCR

Total RNA was extracted from HUVECs using an RNA extraction kit (Qiagen, Germantown, MD). cDNA was generated using the SuperScript II Reverse Transcriptase kit (Invitrogen, Grand Island, NY) and used as a template for 30 cycles of PCR amplification for gene expression analysis using a Thermal Cycler (Bio-Rad). Real-time PCR was carried out with the Mx3000P QPCR System (Agilent Technologies, Santa Clara, CA) using SYBR Green Supermix (Bio-Rad) according to the manufacturer’s instructions. All amplifications were performed in triplicate, and 18S ribosomal RNA (rRNA) was used as the internal control. The primers used for P-selectin were: F-5′-TGAGCACTGCTTGAAGAAAAAGC-3′; R-5′-CACGATTTCACATTCTGGCCC-3′, and for 18S rRNA: F-5′-TTGGAG GGCAAGTCTGGTG-3′; R-5′-CCGCTCCCAAGATCCAACTA-3′.

### Immunofluorescence

Cytospins were prepared from the cell lines, fixed with acetone for 10 minutes at room temperature, and air-dried. For immunofluorescence, cells were incubated with P-selectin polyclonal antibody following incubation with the appropriate (mouse or rabbit) FITC-conjugated secondary antibody (Santa Cruz). Nuclei were counterstained with 4′,6-diamidino-2-phenylindole (DAPI)-containing antifade solution (Vectashield; Vector Laboratories, Burlingame, CA). Fluorescent images were acquired with an EVOS fluorescence microscope (AMGll) and filters specific for FITC and DAPI. Ten different spots were scanned from each slide.

### Cell Adhesion Assays

sRBC adhesion assays were performed using the static gravity adherence assays with dip rinse as described by Hebbel et al. [29] with modifications [12]. Briefly, 80–90% confluent HUVECs were treated with 1 U/mL thrombin (Sigma-Aldrich, St. Louis, MO) or medium alone for 5 minutes before adding sRBCs. After the addition of cells followed by two quick washes, adherent RBCs were stained with Wright Giemsa stains (Sigma-Aldrich) followed by a brief wash with the supplied buffer and counted microscopically in eight randomly selected 0.15 mm^2^ fields. For leukocyte adhesion experiments, assays were performed using the CytoSelect Leukocyte-endothelium adhesion assay kit (Cell Biolabs, San Diego, CA) according to the protocol provided. Briefly, confluent monolayers of transfected HUVECs were seeded onto gelatin-coated 24-well plates in 300 µL basal cell medium supplemented with 2% FBS. Leukocytes were labeled with fluorescent LeukoTracker for 60 minutes at 37°C then added to HUVEC monolayers for 30 minutes. After washing with PBS, cell adhesion was confirmed under the microscope, the cells were treated with the supplied lysis buffer, and fluorescence was measured with a fluorometer (FlexStation II; Molecular Devices) at excitation and emission wavelengths of 485 and 535 nm, respectively.

### Lentivirus Production and Transduction to ECs

Lentiviruses carrying short-hairpin RNAs (shRNAs) against P-selectin were prepared using the LENTI-Smart kit (InvivoGen, San Diego, CA) according to the protocol provided. Briefly, 293T cells with 60–70% confluency were transfected with the supplied plasmids along with 5 µg P-selectin shRNA plasmid (sc-29421-SH, Santa Cruz). The cell culture medium was changed 24 hours after transfection. The medium-containing virus was harvested the next day and passed through a 0.45-µm filter to remove cellular debris. To infect HUVECs, the virus was mixed with an equal volume of fresh medium and protamine sulfate (Sigma-Aldrich) at a final concentration of 10 µg/mL. Cells were washed and incubated with the virus mixture overnight. The next day, cells were fed fresh, virus-free medium. After another 24 hours, puromycin was added at 2.5 µg/mL and infected cells were incubated under selection for 2 weeks before experiments were performed.

### Statistics

The data shown are the means ± SEM. At least three independent experiments were performed for each study. Statistical significance was analyzed using ANOVA between the groups. P values less than 0.05 were considered significant.

## Results

### Role of Glycosylation Inhibitors on P-selectin Expression in HUVECs and Platelets

**Figure 1:**
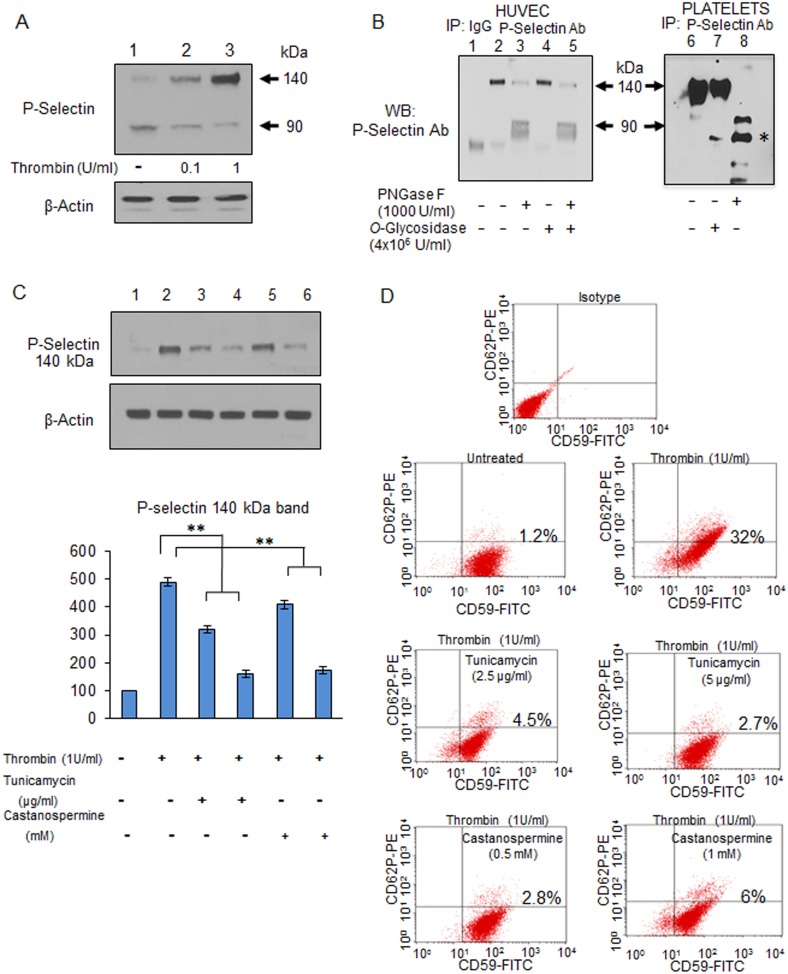
Glycosylation of P-selectin expressed on HUVECs. **A**. Western blotting of HUVECs treated or not treated with 0.1 to 1.0 U/mL thrombin. Lanes: 1, untreated; 2, 0.1 U/mL; 3, 1.0 U/mL. β-Actin was the internal control. Experiments were repeated at least three times and a representative blot was shown. **B**. Digestion by glycosidases of P-selectin prepared from HUVECs (left panel) or platelets (right panel). P-selectin purified by immunoprecipitation was digested for 1 hour with 1000 U/mL PNGase F and/or 4×10^6 ^U/mL *O*-glycosidase and resultant products were analyzed by Western blotting. (Left panel) Lanes: 1, no P-selectin added; 2, P-selectin only; 3, P-selectin digested by PNGase F; 4, P-selectin digested by *O*-glycosidase; 5, P-selectin digested by PNGase and *O*-glycosidase. (Right panel) Lanes: 6, P-selectin only; 7, P-selectin digested by *O*-glycosidase; 8, P-selectin digested by PNGase. The protein band indicated by an asterisk denotes a non-specific band. Ab, antibody; IP, immunoprecipitation: kDa; kilo Dalton; WB, Western blotting. Experiments were repeated three times and a representative blot was shown. **C**. Effects of glycosylation inhibitors on P-selectin expression on HUVECs. Cells were cultured for 48 hours in the presence or absence of tunicamycin or castanospermine at various concentrations followed by stimulation with 1 U/mL thrombin. Cell lysates (30 µg) were subjected to Western blotting analysis. Protein band intensity, which was analyzed by ImageJ v 1.43 software (NIH), was normalized to β-Actin intensity used as a loading control. Lanes: 1, cells without any treatment, 2, cells treated by thrombin only; 3 & 4, cells pretreated with 2.5 µg/mL and 5 µg/mL tunicamycin, respectively, and stimulated by 1 U/mL thrombin; 5 & 6, cells pretreated with 0.5 mM and 1 mM castanospermine, respectively, and stimulated by 1 U/mL thrombin. The intensities of the protein band in lane 1 were set to 100%. **, p<0.001 (compared to lane 2). Experiments were repeated at least three times and a representative blot is shown. **D**. Flow cytometric analysis of P-selectin (CD62P) expression on HUVECs treated with glycosylation inhibitors. Cells were cultured in the absence or presence of tunicamycin (2.5 to 5.0 µg/mL) or castanospermine (0.5 to 1.0 mM) followed by 1 U/mL thrombin treatment. Cells were analyzed by flow cytometry using CD62P antibody conjugated with PE. CD59-FITC was used as the positive marker for ECs. Flow cytometry analyses were repeated at least three times and a representative result was shown.

Although P-selectin expression was analyzed in platelets and HEL cells in detail in a previous study [30], phorbol myristate acetate, a tumor promoter [31], was used to investigate the mechanisms for P-selectin expression. Because SCD is associated with a hypercoagulation state resulting in part from increased levels of the thrombin-antithrombin III complex [32], [33], we used thrombin as a physiological regulator for P-selectin [34]. We first analyzed P-selectin expression on HUVECs by Western blot. Two protein bands were detected at the positions of 90 kDa and 140 kDa (Fig. 1A lane 1). Thrombin treatment of HUVECs increased the intensity of the 140 kDa protein band, but decreased that of the 90 kDa band in a dose-dependent manner (Fig. 1A lanes 2 & 3). Because P-selectin is a 90 kDa protein and highly glycosylated [35], it is likely that the 90 kDa and 140 kDa bands are non-glycosylated and glycosylated forms, respectively [30]. To verify that the 140 kDa band is a glycosylated form, cell lysates prepared from HUVECs treated with thrombin (1 U/mL) and from untreated platelets were immunprecipiated with P-selectin antibody. The immunopurified P-selectin was digested with *N*- and *O*-glycosidases (Fig. 1B). The 140 kDa protein from HUVECs was digested with PNGase F, an endoglycosidase that removes N-linked oligosaccharides [28], and with *O*-glycosidase which releases *O*-linked disaccharides from glycoproteins [36]. In both HUVECs and platelets, the 140 kDa bands were shifted to 90 kDa bands after digestion by PNGase F (Fig. 1B lanes 3 & 8) but not by *O*-glycosidase (Fig. 1B lanes 4 & 7), suggesting that the 140 kDa protein band is a *N*-glycosylated form of the native P-selectin 90 kDa protein.

Next, we examined the effects of glycosylation inhibitors on P-selectin expression in HUVECs treated with 1 U/mL thrombin (Fig. 1C). Here two structurally different glycosylation inhibitors were used. Tunicamycin inhibits the production of *N*-glycans [37], while castanospermine is an indolizine alkaloid which suppresses the activity of alpha-glycosidase [38]. Both tunicamycin and castanospermine reduced expression of the 140 kDa protein band by 60–70%, on HUVECs (Fig. 1C lanes 3 to 6). These results suggest that glycosylation inhibitors may be able to modulate P-selectin expression on HUVECs, as glycosylation is a critical process for the cell surface expression of P-selectin in ECs [24]. Next, we investigated the cell surface expression of P-selectin on HUVECs treated with glycosylation inhibitors by flow cytometry (Fig. 1D). In untreated HUVECs, only 1.2% of the cells expressed P-selectin on the cell surface as determined by flow cytometry analysis, while 5-minute treatment with thrombin (1 U/mL) drastically increased the number of CD62P-positive cells by up to 32% (Fig. 1D). However, the addition of tunicamycin (2.5–5 µg/mL) or castanospermine (0.5–1 mM) to HUVECs treated with thrombin reduced the number of P-selectin-positive HUVECs by 81–92%. Thus, these results indicate that the cell surface expression of P-selectin is efficiently inhibited by glycosylation inhibitors.

### Molecular Mechanisms Underlying P-selectin Inhibition by Glycosylation Inhibitors

**Figure 2:**
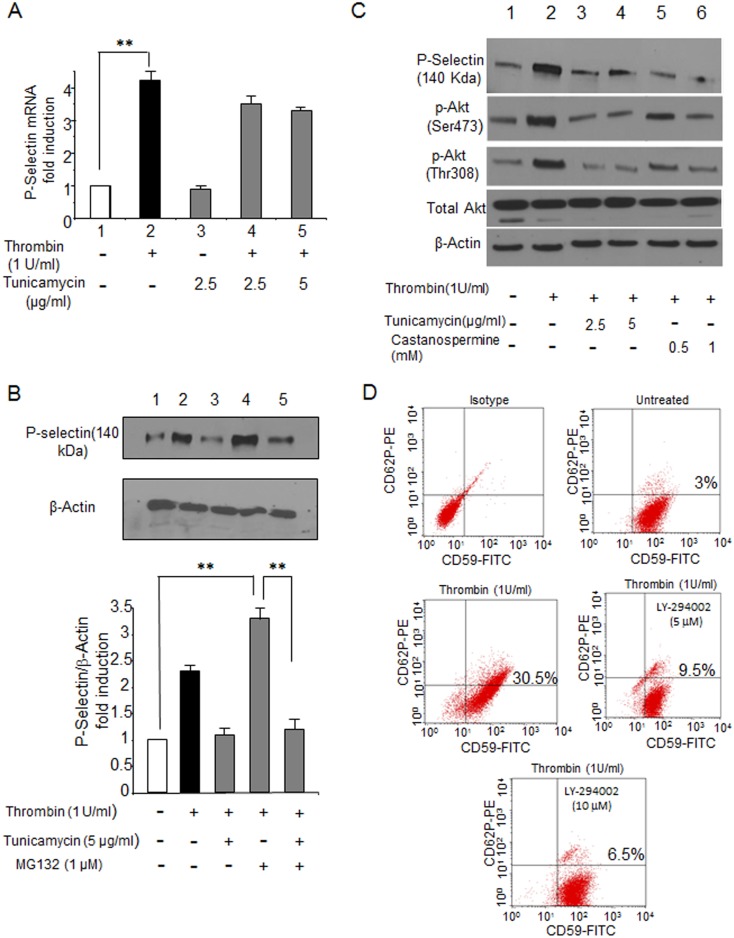
Molecular effects of glycosylation inhibitors on P-selectin mRNA expression (A) and the proteasomal degradation of P-selectin (B) on HUVECs. **A**. Tunicamycin has no effect on P-selectin mRNA levels. HUVECs were incubated with 2.5 or 5 µg/mL tunicamycin for 48 hours before 1 U/mL thrombin was added. Total RNAs were prepared and P-selectin mRNA levels were determined by real time-PCR analysis by using 18S rRNA as an internal control. The P-selectin mRNA level of untreated HUVECs was set to 1. Lanes: 1, untreated HUVECs; 2, thrombin-stimulated HUVECs; 3, HUVECs pretreated with 2.5 µg/mL tunicamycin; 4, HUVECs pretreated with 2.5 µg/mL tunicamycin and then stimulated by 1 U/mL thrombin; 5, HUVECs pretreated with 5 µg/mL tunicamycin and then stimulated by 1 U/mL thrombin. **, p<0.0001. **B**. Effects of tunicamycin on proteasomal degradation of P-selectin. HUVECs were pretreated for 1 hour with or without 1 µM MG132, treated for 48 hours with no, 2.5, or 5 µg/mL tunicamycin, and then treated with or without 1 U/mL thrombin. Cell lysates (30 µg) prepared from HUVECs were analyzed by Western blotting as described in Materials and Methods. β-Actin was used for the loading control. The ratio of the P-selectin protein level to that of β-actin was set to 1. Lanes: 1, untreated HUVECs; 2, thrombin-stimulated HUVECs; 3, HUVECs pretreated with 2.5 µg/mL tunicamycin; 4, HUVECs pretreated with MG132 and then stimulated with thrombin; 5, HUVECs pretreated with MG132, then with 5 µg/mL tunicamycin, and stimulated with thrombin. Experiments were repeated three times that gave similar results and a representative blot is shown. **, p<0.0001 **C**. Effects of glycosylation inhibitors on AKT phosphorylation. HUVECs were cultured for 48 hours in the presence or absence of tunicamycin or castanospermine at various concentrations followed by activation with 1 U/mL thrombin. Western blot analysis was performed to determine the phosphorylation status of AKT (Ser473 and Thr308). β-Actin was the loading control. **D**. AKT inhibitor suppresses CD62P expression on HUVECs. Flow cytometric analysis of P-selectin (CD62P) expression on HUVECs treated with the AKT inhibitor LY-294002 for 1 hour and subsequently stimulated with 1 U/mL thrombin. CD59-FITC was used as the positive marker for ECs. Flow cytometry analyses were repeated three times and a representative result was shown.

We sought to gain further insight into the molecular mechanisms by which tunicamycin downregulates P-selectin expression on the cell surface of HUVECs. Tunicamycin has been shown to regulate gene expression at the mRNA level [39], [40] or through ubiquitin-mediated degradation [41]. First, we determined whether tunicamycin has a regulatory effect on P-selectin mRNA expression on HUVECs (Fig. 2A). Thrombin upregulated P-selectin mRNA levels in HUVECs more than 4-fold, as previously reported [42], but there was no significant difference in the thrombin-induced P-selectin mRNA levels even after pretreatment with tunicamycin (Fig. 2A lanes 3 to 5), indicating that tunicamycin has little effect on the P-selectin mRNA level. Next, we examined whether tunicamycin downregulated P-selectin 140 kDa protein expression by the ubiquitin-proteasome proteolytic pathway (Fig. 2B). Although thrombin treatment enhanced the intensity of the 140 kDa band about 2.5-fold (Fig. 2B lane 2), pretreatment of HUVECs with tunicamycin reduced the expression level to that of untreated cells (Fig. 2B lane 3). Thus, tunicamycin may downregulate thrombin-induced expression of the 140 kDa protein band by inhibiting the glycosylation process or by stimulating the ubiquitin-mediated proteasomal proteolytic pathway, as reported previously [41]. To further investigate the molecular mechanisms by which tunicamycin inhibited expression of the 140 kDa protein band, HUVECs were treated with 1 µM MG132, an inhibitor of proteasomal proteolysis pathway [43], and subsequently with tunicamycin (5 µg/mL) for 48 hours. The pretreatment of HUVECs with MG132 alone augmented the expression of P-selectin on the 140 kDa band by blocking proteasomal pathways (Fig. 2B, lane 4). However, when HUVECs were treated with tunicamycin in the presence of MG132, expression of the P-selectin 140 kDa band was suppressed significantly (Fig. 2B lane 5). These results show that tunicamycin downregulates P-selectin expression by inhibiting glycosylation.

Tunicamycin is known to inhibit the synthesis of *N*-glycans [44]. The cell-surface expression of *N*-glycosylated proteins depends on proper folding and the degree of *N*-glycan branching [45]; the phosphoinositide 3 kinase (PI3K)/AKT pathway is involved in the regulation of such glycosylation-relevant processes [46]. Here we examined whether phosphorylation of AKT on HUVECs is affected by glycosylation inhibitors, as the activity of the PI3K/AKT pathway depends in part on the phosphorylation at Ser473 and Thr308 of AKT. Tunicamycin and castanospermine downregulated phosphorylation on both Ser473 and Thr308 on HUVECs (Fig. 2C). To verify that the PI3K/AKT pathway is involved in P-selectin expression on the cell surface of HUVECs, we analyzed CD62P expression by flow cytometry. HUVECs treated with the AKT inhibitor LY-294002 had a dramatic reduction in CD62P expression (Fig. 2D), suggesting that the PI3K/AKT pathway is involved in P-selectin expression on HUVECs.

### Glycosylation Inhibitors Block Hypoxia-induced sRBC and Leukocyte Adhesion to ECs

**Figure 3:**
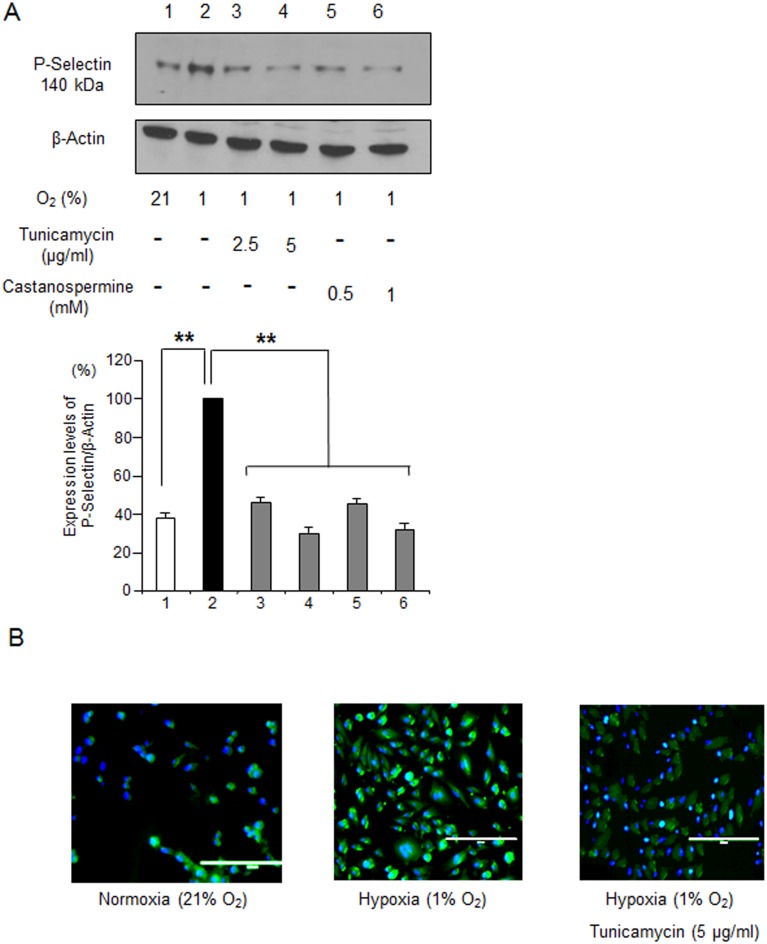
Glycosylation inhibitors block hypoxia-induced P-selectin expression in HUVECs. P-selectin expression in HUVECs exposed to 1% O_2_ was examined by Western blotting (A) and immunofluorescence (B). **A**. Western blotting analysis of P-selectin expression in HUVECs exposed to hypoxia. HUVECs were cultured in the absence or presence of 2.5 or 5.0 µg/mL tunicamycin or 0.5 to 1.0 mM castanospermine, treated with 1 U/mL thrombin, and then were exposed to 1% O_2_ for 1 hour. Cell lysates (30 µg) prepared from HUVECs were analyzed by Western blotting as described in Materials and Methods. β-Actin was as the loading control. The ratio of the P-selectin protein level to that of β-actin was set to 100%. Note that this experiment focused on the expression of the 140 kDa band in response to hypoxia. **, p<0.0001 **B**. P-selectin expression on HUVECs by immunohistochemistry. HUVECs were permeabilized and fixed as described in Materials and Methods. Note that expression of P-selectin (green) was induced by exposure to 1-hour hypoxia (1% O_2_), but by pretreatment of 5 µg/mL tunicamycin.

Thrombin is a strong chemical inducer of P-selectin expression [42]. Hypoxic stress, by comparison, is a physiological insult that induces vaso-occlusive crisis in patients with SCD [47]. Hypoxia has been shown to induce P-selectin expression [48] and as such is more of a physiological inducer of P-selectin. To further investigate whether glycosylation inhibitors can inhibit hypoxia-induced P-selectin expression, HUVECs were incubated under hypoxia of 1% O_2_ for 1 hour in the absence and presence of glycosylation inhibitors. P-selectin expression was analyzed by Western blot (Fig. 3A). P-selectin expression levels increased about 2.5-fold in response to hypoxia (Fig. 3A lanes 1 & 2), however, adding tunicamycin or castanospermine to the cultures suppressed HUVEC P-selectin expression to levels comparable to that of HUVECs at normoxia (Fig. 3A lanes 3 to 6). These results were confirmed by immunofluorescence (Fig. 3B). Thus, glycosylation inhibitors efficiently inhibited P-selectin expression that had been elevated by hypoxic conditions.

**Figure 4:**
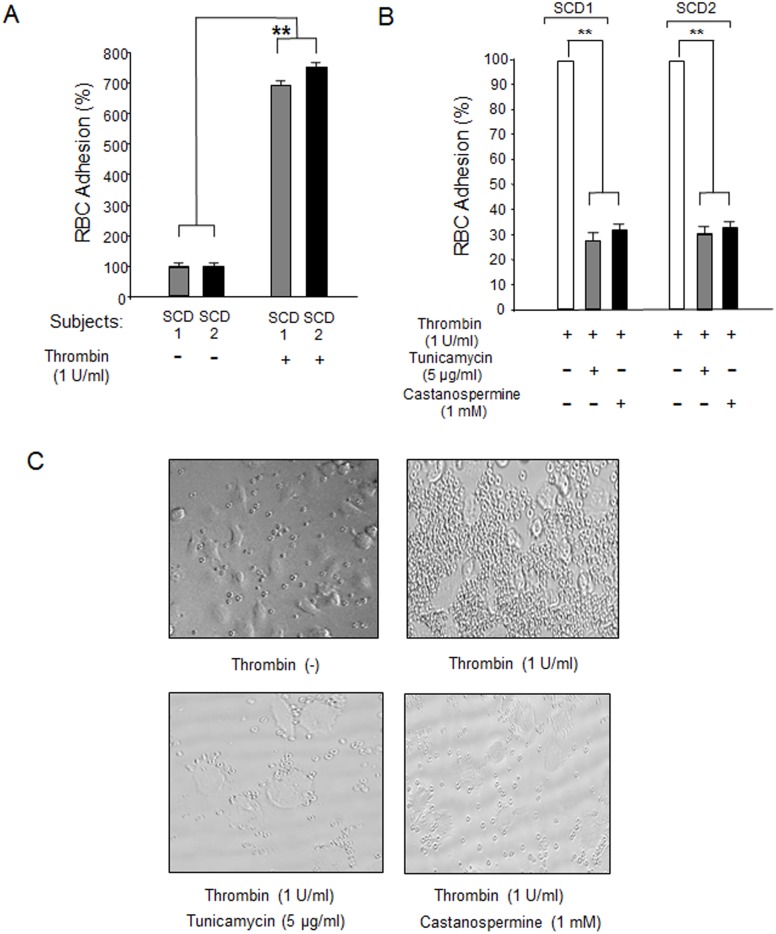
Glycosylation inhibitors block thrombin-induced sRBC adhesion to HUVECs. **A**. Thrombin induces sRBC adhesion to HUVECs. sRBCs were prepared from two sickle cell model mice (SCD 1 & SCD 2) [26]. HUVECs were treated with 1 U/mL thrombin for 5 minutes after which sRBCs were added. The level of sRBC adhesion to untreated HUVECs was set to 100%. **, p<0.0001 **B**. Glycosylation inhibitors decrease thrombin-induced sRBC adhesion to HUVECs. HUVECs were pretreated with glycosylation inhibitors for 48 hours and P-selectin expression was stimulated by 1 U/mL thrombin. sRBCs were then added to HUVECs for adhesion assays. The levels of sRBC adhesion to HUVECs that were not treated with glycosylation inhibitor but stimulated with thrombin were set to 100%. **, p<0.0001 **C**. Representative microscopic images (x10 magnification) showing sRBC adhesion to HUVECs. Note that both tunicamycin and castanospermine inhibit sRBC adhesion to thrombin-activated HUVECs.

**Figure 5:**
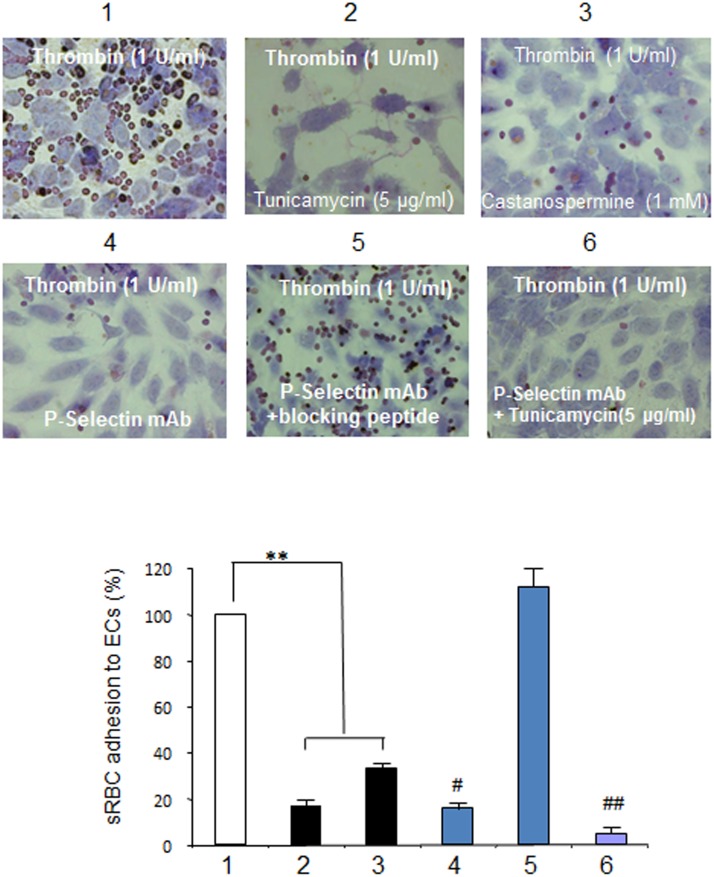
Comparison of sRBC adhesion inhibition by glycosylation inhibitors and P-selectin monoclonal antibody. HUVECs that were cultured as described in Materials and Methods were pretreated with saline or glycosylation inhibitors for 48 hours and stimulated with 1/mL thrombin. sRBCs were isolated from SCD patients and added to HUVECs. To examine the inhibition of sRBC adhesion, P-selectin monoclonal antibody was added to HUVECs prior to the addition of sRBCs. To inactivate P-selectin monoclonal antibody, the antibody was premixed with blocking peptide. Lanes: 1, HUVECs stimulated with 1U/mL thrombin; 2, HUVECs pretreated with 5 µg/mL tunicamycin; 3, HUVECs pretreated with 1 mM castanospermine; 4, HUVECs added with P-selectin monoclonal antibody; 5, HUVECs added with P-selectin monoclonal antibody premixed with blocking peptide; 6, HUVECs pretreated with 5 µg/mL tunicamycin and further added with P-selectin monoclonal antibody. **, p<0.0001; ^##^, p<0.01 (vs lanes 2 & 4).

**Figure 6:**
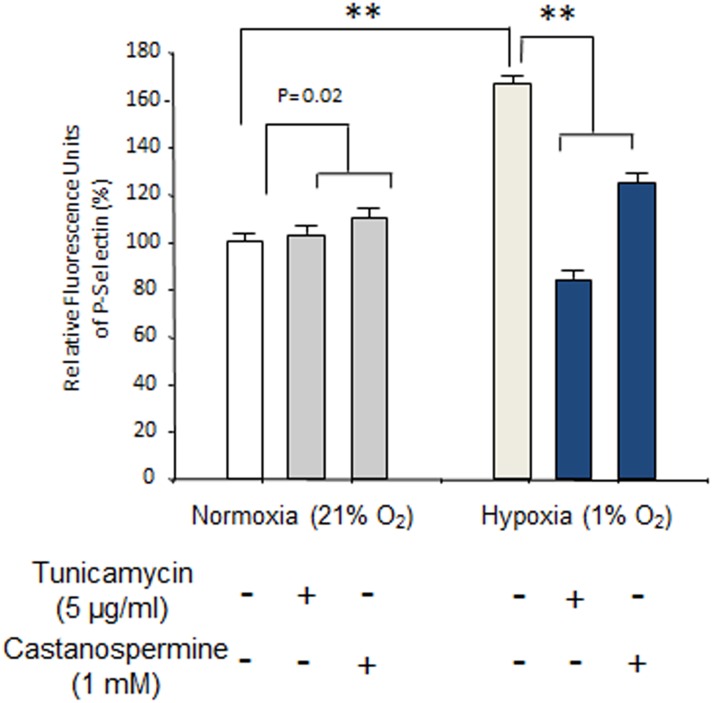
Effect of glycosylation inhibitors on hypoxia-induced leukocyte adhesion to HUVECs. Leukocytes were isolated from SCD model mice. HUVECs pretreated with glycosylation inhibitors were exposed to normoxia (21% O_2_) or hypoxia (1% O_2_). The adhesion levels of leukocytes to HUVECs at normoxia was set to 100%. Data are mean ± SEM; n = 3 to 5. **, p<0.0001.

Our recent in vivo studies using intravital microscopy demonstrated a critical role for P-selectin in sRBC adhesion to ECs [21], [49]. To investigate the effect of glycosylation inhibitors on sRBC adhesion, we performed in vitro sRBC adhesion assays using HUVECs (Fig. 4). We first isolated sRBCs from two SCD model mice [26] and examined sRBC adhesion to HUVECs as described in Materials and Methods. The adhesion of sRBC to HUVECs was markedly enhanced when HUVECs were treated with 1 U/mL thrombin (Fig. 4A), a result consistent with the enhancement of P-selectin expression by thrombin (Fig. 1A). In contrast, pretreating HUVECs with glycosylation inhibitors such as tunicamycin and castanospermine drastically reduced sRBC adhesion (Fig. 4B; see Fig. 4C for photomicrographs of sRBC adhesion to HUVECs treated with thrombin in the presence or absence of glycosylation inhibitors). We next examined whether glycosylation inhibitors of sRBC adhesion were comparable with that of anti-P-selectin monoclonal antibody, which was a control P-selectin inhibitor in our previous in vivo study [21]. Here we prepared sRBCs from SCD patients who were homozygous for the β^s^ mutation. The sRBC adhesion score of HUVECs pretreated with 5 µg/mL tunicamycin was less than 20% of untreated HUVECs mixed with sRBCs (Fig. 5 lanes 1 & 2), and castanospermine produced a slightly higher sRBC adhesion score (lane 3). Interestingly, the adhesion score of HUVECs treated with anti-P-selectin monoclonal antibody was not statistically significant from that of HUVECs pretreated with tunicamycin (lanes 2 & 4), demonstrating that tunicamycin is as effective as anti-P-selectin antibody in preventing sRBC adhesion to ECs. Combining glycosylation inhibitors with an anti-P-selectin antibody was more efficacious than either agent alone in inhibiting sRBC adhesion to HUVECs (lane 6). Like sRBCs, leukocytes also have a role in initiating vaso-occlusion in SCD [20]. To examine the effects of glycosylation inhibitors on leukocyte adhesion (Fig. 6), HUVECs were treated with 5 µg/mL tunicamycin or 1 mM castanospermine then exposed to either normoxia of 21% O_2_ or hypoxic stress of 1% O_2_ for 1 hour. Under normoxia, leukocyte adhesion scores did not decrease on HUVECs pretreated with glycosylation inhibitors (Fig. 6 left columns). In contrast, adhesion scores increased by about 70% in response to hypoxia, while treatment with tunicamycin and castanospermine decreased leukocyte adhesion scores by 48% and 24%, respectively (Fig. 6 right columns). Thus, glycosylation inhibitors effectively reduced leukocyte adhesion to ECs under hypoxia.

**Figure 7:**
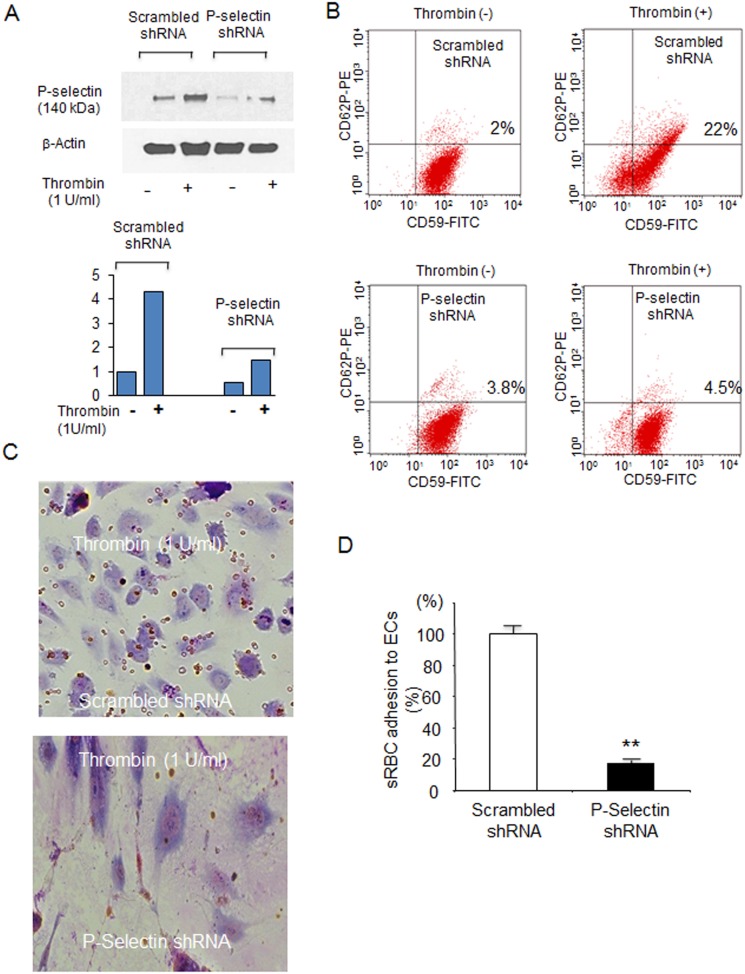
Legitimate role of P-selectin in thrombin-induced sRBC adhesion to HUVECs. **A & B**. shRNA-mediated knock-down of P-selectin in HUVECs. Cells were infected with lentivirus carrying shRNA constructs against P-selectin. The effect of P-selectin shRNAs on thrombin-induced P-selectin expression in HUVECs was verified by Western blotting (A) and flow cytometry (B). The levels of P-selectin expression in HUVECs were quantified by ImageJ software (NIH) (right panel of **A**). **C & D**. Effect of shRNA-directed P-selectin knockdown on sRBC adhesion to thrombin-stimulated HUVEC. **C**. Pictures are representative optical fields (×10 magnification) of sRBC adhesion to HUVEC. The results of sRBC adhesion was summarized in **D**. **, p<0.0001.

Glycosylation inhibitors may modulate the expression of a variety of proteins [37] including E-selectin [50]. To verify that a decrease in P-selectin expression by glycosylation inhibitors does in fact reduce sRBC adhesion to HUVECs, we used shRNAs to suppress P-selectin expression on HUVECs and examined sRBC adhesion. HUVECs were subjected to shRNA-mediated knockdown of P-selectin. P-selectin expression on HUVECs treated with shRNAs was reduced on Western blot (Fig. 7A see right panel for summary) and flow cytometric analysis (Fig. 7B). Although P-selectin expression was induced on HUVECs transfected with scrambled shRNAs in response to thrombin, no induction was seen on HUVECs transfected with P-selectin-specific shRNAs (Fig. 7A). Similarly, thrombin treatment increased the number of CD62P-positive HUVECs from 2% to 22%, but no significant increase was observed with HUVECs transfected with P-selectin-specific shRNAs (Fig. 7B). Of note, the thrombin-induced sRBC adhesion to HUVECs that were treated with P-selectin shRNAs was suppressed by more than 90% in comparison with HUVECs transfected with scrambled shRNAs (Fig. 7C & D). These results clearly demonstrate that the reduction in sRBC adhesion on HUVECs treated with glycosylation inhibitors is solely dependent on reduced P-selectin expression.

## Discussion

Although multiple adhesion molecules have been shown to regulate adhesive interactions among various types of cells in both in vitro and ex vivo systems [12], [51], [52], recent intravital microscopic studies have demonstrated that P-selectin plays a significant role in the adhesion of sRBCs and leukocytes to ECs [13], [21], [53], an involvement that was also verified using P-selectin knock-out mice [54]. Potential therapeutics targeting P-selectin include nitric oxide [55], LMWHs [22], [56], and an anti-P-selectin aptamer [21]. Patient compliance is a concern with both LMWHs and the aptamer because they must be infused intravenously to block cell adhesion. As noted, a recent clinical trial of a P-selectin inhibitor failed to reduce daily pain scores in SCD patients [23], suggesting a need for novel cell adhesion inhibitors.

We sought to identify new P-selectin inhibitors by building upon experimental evidence that P-selectin is a heavily glycosylated protein in multiple cell types [30]. Because glycosyltrasferase expression levels vary significantly depending on cell type [57], we initially examined P-selectin molecules expressed in ECs. Both non-glycosylated and glycosylated forms were expressed in steady-state ECs which migrated to 90 kDa and 140 kDa, respectively (Fig. 1A lane 1). In contrast, the glycosylated 140 kDa form is dominant in platelets (Fig. 1B lane 6) [30]. As reported previously [30], we found that the 140 kDa form is induced by thrombin (Fig. 1A lanes 2 & 3), a P-selectin inducer [42], and that *N*-glycosylation, but not *O*-glycosylation, plays a role in P-selectin glycosylation (Fig. 1B lanes 3 & 4). Consistent with these in vitro studies, the *N*-glycosylation inhibitor tunicamycin and α-glycosidase inhibitor castanospermine [38] efficiently inhibited expression of the 140 kDa form in cytoplasm (Fig. 1C) and the cell surface expression of CD62P of ECs (Fig. 1D). It is striking that a small reduction in expression of the 140 kDa form by a glycosylation inhibitor results in such a strong suppression of the cell surface expression of P-selectin (CD62P) on ECs (see results in Fig. 1C & D), suggesting that glycosylation inhibitors could inhibit cell adhesion at low concentrations. Furthermore, as to the molecular action of tunicamycin, our studies showed that neither transcriptional mechanisms or proteasomal degradations are responsible for the reduction of P-selectin expression on HUVECs treated with tunicamycin (Fig. 2A & B), supporting the notion that tunicamycin suppresses P-selectin expression by inhibiting glycosylation. Importantly, both tunicamycin and castanospermine inhibited hypoxia-induced P-selectin expression in ECs (Fig. 3A & B); hypoxia is believed to be involved in the mechanisms underlying elevated expression of P-selectin in SCD [12], [58]. These results demonstrate that glycosylation inhibitors may be capable of inhibiting P-selectin expression in ECs.

The current study has revealed a novel molecular link between tunicamycin and an intracellular signaling pathway. Tunicamycin blocks the synthesis of N-linked glycoproteins by inhibiting GlcNAc phosphotransferase [59]. The PI3K/AKT pathway is shown to regulate protein folding and N-glycan branching [46]. We found that treating HUVECs with both tunicamycin and castanospermine downregulated phosphorylation of AKT (Fig. 2C & D). That glycosylation inhibitors were able to decrease phosphorylation of AKT suggests that either 1) glycosylation inhibitors may regulate AKT activity directly by dephosphorylation or 2) inhibiting the glycosylation process may provide negative feedback to AKT activity by regulating phosphorylation because the PI3K/AKT pathway is involved in the synthesis of N-glycans [46]. Our study using the PI3K inhibitor LY294002 [60] showed that the PI3K/AKT pathway is involved in P-selectin regulation in ECs (Fig. 2D). It would be interesting to examine the mechanisms by which glycosylation inhibitors regulate AKT phosphorylation.

To confirm that glycosylation inhibitors are efficacious as cell adhesion inhibitors, we performed in vitro cell adhesion assays using HUVECs [12], [29]. Thrombin-induced sRBC adhesion to HUVECs was suppressed by more than 60% when tunicamycin or castanospermine was added (Fig. 4A & B), and the degree of sRBC inhibition by glycosylation inhibitors was as high as P-selectin antibody (Fig. 5), which was the most effective P-selectin inhibitor used in our intravital microscopic studies [21], [49]. Both tunicamycin and castanospermine exerted an inhibitory effect on leukocyte adhesion to ECs as well (Fig. 6). Furthermore, our shRNA study confirmed that reducing P-selectin expression on ECs markedly inhibited the thrombin-induced adhesion of sRBCs to HUVECs, suggesting that a reduction in the expression of P-selectin, but not other adhesion molecules, is sufficient to inhibit sRBC adhesion to ECs (Fig. 7). Together with the fact that a number of glycosylation inhibitors are currently used for various clinical disorders [59], this study demonstrates that glycosylation inhibitors may represent a novel class of adhesion inhibitors for sRBCs and leukocytes and might be useful in the treatment of SCD.

Other anti-adhesive compounds currently under investigation as treatments for SCD include human immunoglobulin [61], monoclonal antibody to αVβ3 [52], and LMWHs [22]. The renal toxicity of human immunoglobulin has raised concerns [62], as have the inadequate pharmacokinetics, tissue accessibility, and immunogenicity of therapeutic antibodies. In addition, antibodies and LMWHs have to be injected intravenously to achieve optimal clinical efficacy [63], which may affect patient compliance. In contrast, glycosylation inhibitors are as efficient as P-selectin in limiting cell adhesion (Fig. 5A) and are devoid of immunogenicity. However, because of potential toxicities, we must use caution as glycosylation of proteins and lipids is a critical post-translational modification to maintain the functional roles of proteins or lipids [25]. It is encouraging that treating HUVECs with 5 µg/mL tunicamycin did not significantly reduce cell viability (results not shown) and high doses of castanospermine showed little toxicity in mice [64], [65]. Recently the effects of glycosylation inhibitors have been tested in multiple clinical disorders including diabetes, cancer, and viral infections such as human immunodeficiency virus and hepatitis B [25], [37]. Interestingly, even a small decrease in the expression of the 140 kDa component of P-selectin by tunicamycin resulted in a strong inhibition on the cell surface expression of CD62P (Fig. 1C & D), thereby efficiently suppressing the adhesion of sRBCs to ECs (Fig. 5), suggesting a strong inhibitory effect of glycosylation inhibitors on cell adhesion. Glycosylation inhibitors could possibly be administered to SCD patients with negligible adverse effects.

In fact, glycosylation inhibitors may have an advantage as a cell adhesion inhibitor over other anti-adhesive therapeutics. Miglitol, an alpha-glycosidase inhibitor like castanospermine, has been used to treat type 2 diabetes for nearly two decades [66], indicating the longer term clinical safety of alpha-glycosidase inhibitors. Moreover, the anti-inflammatory effects of miglitol reduce C-reactive protein levels [67] and may help to alleviate SCD-related inflammation. In addition, glycosylation inhibitors may be able to inhibit the expression of other adhesion molecules such as E-selectin [50] which plays a role in leukocyte adhesion in SCD model mice [19], and VCAM-1, which is a glycoprotein involved in cell adhesion in SCD [12].

The unexpected outcome of the PPS clinical trial [23] provided insight into the mechanisms underlying SCD pathophysiology. First, the improved microvascular blood circulation and reduced plasma VCAM-1 levels in patients treated with PPS may not be pertinent to the mechanisms that regulate pain in SCD patients or, at the least, may not be sufficient to reduce daily pain scores. Second, although therapeutics targeting P-selectin efficiently inhibit cell adhesion of sRBCs and leukocytes to ECs, as observed by in vitro and in vivo mouse studies [13], [21], the inhibition of cell adhesion may not be germane to or may be inadequate for the reduction in daily pain in steady-state SCD patients. Importantly, this class of chemicals appears to have little effect on P-selectin expression on ECs. Also, PPS and anti-P-selectin aptamer both bind to P-selectin with high affinity, however P-selectin molecules expressed on the cell surface will be promptly internalized to the cytoplasm by endocytosis [35] and the complexes between P-selectin-binding molecules and P-selectin disappear quickly from the cell surface. Removal of P-selectin and PPS complexes by internalization would consume PPS molecules quickly and the number of PPS molecules available in peripheral blood can be reduced. This could hamper the clinical efficacy of these P-selectin-binding compounds. In contrast, glycosylation inhibitors are capable of almost completely suppressing P-selectin expression on ECs (Fig. 1D) and such reduction on the cell surface may be important in the regulation of daily pain scores of SCD patients. This view is supported by a study which reported that nitric oxide decreased P-selectin expression in vitro and in vivo [55] and by our clinical trial in which nitric oxide reduced pain scores in SCD patients with vaso-occlusive crisis [68]. In addition, multiple independent studies had shown substantial clinical effects of nitric oxide breathing on the pathophysiology of SCD [69], [70], [71] and other pulmonary disorders [72]. These lines of basic and clinical evidence suggest that glycosylation inhibitors could prove to be potential therapeutics for SCD patients. More importantly, the expression levels of unglycosylated 90 kDa protein was not suppressed but increased in HUVECs treated with 5 µg/mL tunicamycin for up to 16 hours (data not shown), suggesting that glycosylation inhibitors may not be detrimental to protein synthesis in cells.

## Conclusions

In conclusion, our study results clearly demonstrate the role of two structurally distinct glycosylation inhibitors, tunicamycin and castanospermine, in inhibiting the adhesion of sRBCs and leukocytes to ECs. In contrast to other anti-adhesive compounds, glycosylation inhibitors appear to efficiently suppress P-selectin expression on ECs without unfavorable effects on cell integrity including protein synthesis in ECs. Glycosylation inhibitors may represent a novel class of cell adhesion inhibitors. Alpha-glycosidase inhibitors such as castanospermine derivatives [64] and miglitol have been used in disorders such as diabetes and more new compounds are under development to inhibit such viral infections as human immunodeficiency virus and hepatitis B [25]. Further studies to investigate the in vivo effects of glycosylation inhibitors on cell adhesion include intravital microscopy, the state-of-the art technique used to assess cell adhesion [21], [49].
